# Medial-Pivot Total Knee Arthroplasty with Kinematic Alignment Produces Similar Clinical Outcomes in Valgus and Varus Deformities

**DOI:** 10.2106/JBJS.OA.25.00308

**Published:** 2026-01-13

**Authors:** Emma N. Horton, Lauren K. Holbrook, David F. Scott

**Affiliations:** 1 Spokane Joint Replacement Center, Spokane, Washington; 2 Elson S. Floyd College of Medicine Washington State University, Spokane, Washington

## Abstract

**Background:**

Mechanical alignment total knee arthroplasty (TKA) in valgus knees requires soft tissue releases and complex techniques that are unnecessary with kinematic alignment (KA). Few studies have evaluated the outcomes of KA in valgus knees, and fewer have studied KA with medial-pivot (MP) implants. This study compared the clinical outcomes of KA-TKA with MP implants (KA/MP-TKA) in patients with preoperative varus versus valgus alignment. We hypothesized that outcomes following KA/MP-TKA would be equivalent.

**Methods:**

A prospective database was queried to identify patients who underwent primary KA-TKA with MP implants. Patients were included if they had a preoperative hip-knee-ankle angle ≤177° (varus, n = 302) or ≥183° (valgus, n = 51). A total of 353 consecutive patients were identified. The Forgotten Joint Score (FJS), Knee Injury and Osteoarthritis Outcome Score (KOOS-JR), Knee Society Score (KSS), and range of motion were collected preoperatively and at 6-week, 6-month, and 1-year visits.

**Results:**

Patients with preoperative valgus alignment had worse KSS Function and KOOS-JR scores preoperatively (p = 0.04 and p = 0.02, respectively); all other baseline outcomes were comparable. Postoperatively, the valgus group demonstrated better KOOS-JR at 6 weeks (p = 0.004), KSS Pain-Motion at 6 months (p = 0.02), and FJS at 1 year (p = 0.03). The varus group showed better KSS Function at all postoperative visits (p < 0.02). There were no statistically significant differences in flexion or extension between the groups.

**Conclusions:**

Patients with valgus alignment undergoing KA/MP-TKA achieved postoperative outcomes that were clinically equivalent or superior to those of patients with varus alignment. These findings support KA-TKA using MP implants as an appropriate surgical approach for preoperative valgus alignment.

## Introduction

Preoperative valgus knee alignment affects 10% to 15% of primary total knee arthroplasty (TKA) cases and occurs 2 to 6 times more frequently in women^1-4^. Anatomic features associated with preoperative valgus alignment—such as hypoplastic lateral femoral condyles, contracted lateral capsules, and medial collateral ligament laxity—may complicate intraoperative balancing and postoperative stability^5-7^. Recommendations vary, with some minimizing bone resection from the lateral condyle due to perceived bone loss and others reporting no clinically significant asymmetry between medial and lateral femoral condyles^1,7,8^.

Mechanical alignment (MA) remains a common TKA approach for valgus knees, yet achieving neutral mechanical alignment can be technically challenging^2,6,9^. There is no consensus on the optimal technique for the soft tissue releases required to correct ligamentous imbalance in MA^2,10-13^, and these soft tissue releases may not correct gap imbalances^9,14^. These techniques include Insall's pie-crust technique, Ranawat's transverse posterolateral capsule division, and Whiteside's progressive release strategy^15-18^.

Although these complex techniques aim to restore balance, they may lead to suboptimal outcomes, instability, prolonged operating room time, and higher complication rates^1,5,19^. Evidence demonstrating the superiority of any technique is limited and subjective^1,3,20^. The variability between surgeons and subjective nature of manual soft tissue assessments compounds this challenge of soft tissue balancing^20^, raising concerns about reproducibility.

Kinematic alignment (KA) is an alternative that aims to replicate the obliquity of the prearthritic joint line and minimize soft tissue releases^21^. Unlike MA, which requires soft tissue releases and unequal bony resections for all patients to achieve a neutral mechanical axis, KA preserves each patient's native geometry^22^. KA may be beneficial for valgus knees because it adapts to their unique anatomy and preserves ligament function. By maintaining natural joint geometry and soft tissue integrity, KA mitigates challenges associated with MA in valgus knees, potentially improving outcomes. Nevertheless, concerns persist that KA-TKA may leave valgus knees with residual malalignment, potentially leading to secondary instability and a less desirable cosmetic appearance^3,9^.

Most TKA implants are engineered for MA compatibility, prioritizing component positioning perpendicular to the mechanical axis to achieve even load distribution to prevent excessive wear and premature loosening^14^. However, these implants may be suboptimal for KA, which restores native joint line obliquity and asymmetric compartment loads, leading to concerns about survivorship^23,24^. Medial-pivot (MP) implant design mimics natural knee kinematics, better aligning with the goals of KA. MP designs feature a highly congruent medial compartment with a ball-and-socket joint, providing superior sagittal plane stability and outcomes compared with posterior-stabilizing and condylar-stabilizing implants in KA-TKA, while the lateral compartment allows natural femoral rollback^25-28^. However, studies evaluating the efficacy of KA/MP-TKA in valgus knees are limited^5^. This study investigated unrestricted KA-TKA using MP implants in patients with preoperative valgus alignment and hypothesized that these patients would achieve postoperative outcomes equivalent to those with preoperative varus alignment.

## Methods

The WCG Institutional Review Board approved the prospective collection of clinical outcomes data for TKA patients enrolled in a single-surgeon, single-site institutional registry study.

### Patient Selection

All TKA patients with primary osteoarthritis consecutively enrolled in a prospective institutional registry between April 2020 and April 2025 were eligible for inclusion (n = 424). Patients with neutral or near-neutral preoperative alignment, defined as a hip-knee-ankle (HKA) angle between 178° and 182° (n = 71), were excluded. The remaining patients (n = 353) were categorized as either varus (HKA ≤177°, n = 302) or valgus (HKA ≥183°, n = 51). No patients were excluded for incompetent medial collateral ligaments.

Within the valgus group, 32 were female and 19 were male. For the varus group, 116 were female and 186 were male (Table I). The women in the valgus group were older (70.7 vs. 67.2, p = 0.02) and had a lower body mass index (BMI) (31.0 vs. 33.0, p = 0.05) than women in the varus group. The mean BMI and age did not significantly differ between the male or combined groups (p = 0.08-0.93) (Table I).

**TABLE I:** Mean (Range) and SD for Demographic Data

Variable	Group	Varus	Valgus	p
Sex	Male	n = 186	n = 19	**0.001**
	Female	n = 116	n = 32	
Age	All	67.7 (42-89) SD: 8.3	69.6 (43-81) SD: 8.5	0.12
	Male	68.0 (42-88) SD: 8.5	67.8 (43-81) SD: 10.8	0.93
	Female	67.2 (48-89) SD: 7.8	70.7 (54-81) SD: 6.8	**0.02**
BMI	All	32.6 (21-48) SD: 4.8	31.3 (23-45) SD: 4.7	0.08
	Male	32.2 (22-48) SD: 4.6	31.8 (23-37) SD: 4.0	0.69
	Female	33.0 (21-44) SD: 5.1	31.0 (23-45) SD: 5.1	**0.05**

BMI = body mass index = kg/m^2^, and SD = standard deviation.

Statistical significance was determined using chi-square for sex distribution and Student's *t*-test for continuous variables; significant p-values are denoted in bold.

### Operative Technique

The principal investigator performed all TKAs using unrestricted caliper-verified KA with manual instrumentation, medial parapatellar arthrotomy, a tourniquet, and cementation of all components^25,29,30^.

The femur was positioned with neutral rotation relative to the posterior condyles. The target resection thickness was defined as equal to the thickness of the prosthetic femoral condyle minus 1 mm for bone removal due to the kerf of the saw blade. Adjustments were made for cartilage loss, assuming 2-mm normal cartilage thickness. The thicknesses of the distal and posterior condyles of the true “ball-in-socket” MP implant device used in all cases were 9 mm and 8 mm, respectively (GMK Sphere, Medacta International). Therefore, the target for the distal resection was 8 mm for unworn cartilage and 6 mm for worn cartilage, while the target for the posterior resection was 7 mm for unworn and 5 mm for worn. If there was partial-thickness cartilage loss, a ring curette was used to scrape the remaining cartilage down to bone. No ligament releases were performed.

### Clinical and Radiographic Evaluation

Preoperative and 1-year postoperative HKA angles were manually measured from long-standing, weight-bearing radiographs. Two independent readers assessed the HKA angles. The primary reader's measurements were used for analysis. The inter-rater reliability intraclass correlation coefficient (ICC) was high (ICC = 0.986).

The principal investigator measured range of motion (ROM) using a large goniometer with the patient sitting. ROM and patient-reported outcome measures (PROMs) were collected at preoperative, 6-week, 6-month, and 1-year visits. PROMs included the Forgotten Joint Score (FJS), Knee Injury and Osteoarthritis Outcome Score (KOOS-JR), and Knee Society Score (KSS).

### Data Analysis

This study used Microsoft Excel (Mac version 16.89), R (R Foundation for Statistical Computing), and Ortho Research Master, an electronic data capture and analysis platform (Spokane Joint Replacement Center, Inc., Spokane). Student's *t*-test and χ^2^ with significance levels of p ≤ 0.05 were used to compare mean scores and categorical variables, respectively, between the groups at each visit.

For PROMs with significant intergroup baseline differences, the mean improvement was calculated as the difference between each postoperative and preoperative mean score. The mean improvements were compared between groups using Student's *t*-test with statistical significance of p ≤ 0.05.

## Results

**Fig. 1:**
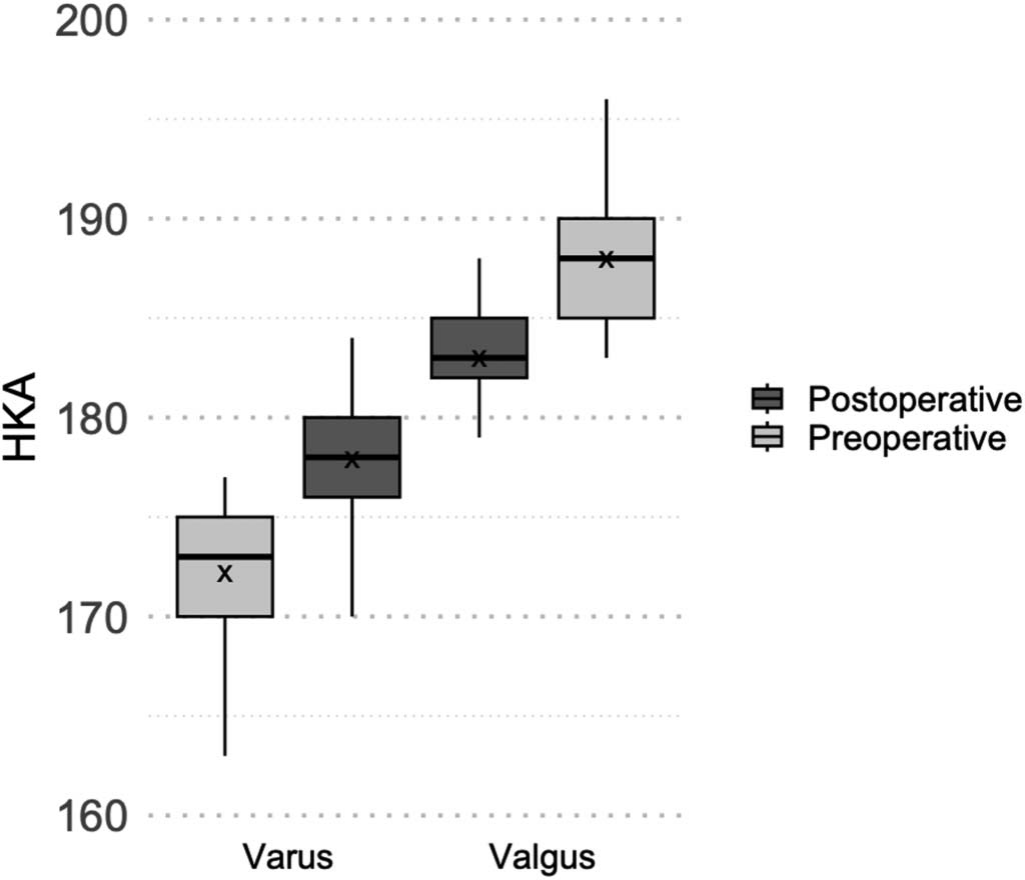
Box-and-whisker plot illustrating the distribution of HKA angles for our designated varus and valgus patient groups, shown preoperatively and postoperatively. Boxes represent the interquartile range, horizontal lines indicate medians, and “X” denotes group means. HKA = hip-knee-ankle.

The valgus group had a mean preoperative HKA angle of 188.0° (range 183-196°), while the varus group had a mean angle of 172.2° (range 160-177°). At 1-year postoperative visit, the valgus group had a mean HKA angle of 183.0° (range 174-190°), while the varus group had a mean angle of 177.9° (range 170-190°) (Fig. 1). Five valgus patients (10%) crossed over to any degree of varus alignment (<180°), and 4 valgus subjects (8%) crossed over to varus as defined in the manuscript (≤177°). In addition, 49 varus subjects (12%) crossed over to any degree of valgus (>180°), and only 18 varus subjects (6%) crossed over to valgus as defined by the manuscript ≥183°. Tourniquet time did not significantly differ between groups (p = 0.21).

**Fig. 2:**
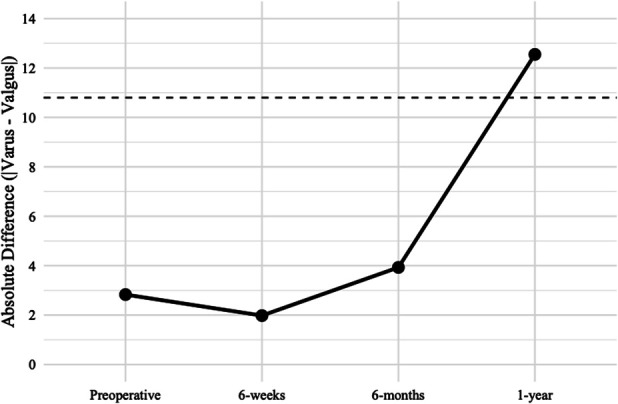
The absolute difference between the mean FJS for varus and valgus cohorts at each time point. Minimal Clinically Important Difference, set at 10.8, is represented by the dotted line. FJS = Forgotten Joint Score.

Patients with preoperative valgus knee alignment had worse KOOS-JR and KSS Function preoperatively (p = 0.02 and *p* = 0.04, respectively). All other preoperative PROMs were equivalent (p > 0.05). The valgus group demonstrated a statistically better FJS at 1 year (p = 0.03; Fig. 2), KOOS-JR at 6 weeks (p = 0.004), and KSS Pain-Motion at 6 months (p = 0.02). The varus group demonstrated a statistically better KSS Function at all follow-up visits (p = 0.004-0.017). No other postoperative PROMs significantly differed (p > 0.05). ROM did not differ at any visit (*p* = 0.090-0.994), with the varus and valgus groups averaging 131° and 132° of flexion, respectively, 1 year post-TKA (Table II).

**TABLE II:** Mean (Range) and SD for Patient-Reported Outcome Measures and Range of Motion at the Preoperative Visit and Postoperative Follow-up Visits at 6 Weeks, 6 Months, and 1 Year

Outcome Measure	Varus	Valgus	p
KOOS-JR			
Preoperative	50.56 (0-92) SD: 13.2	45.76 (0-68.28) SD: 12.4	**0.016**
6 weeks	64.37 (28.3-100) SD: 10.2	69.22 (47.5-100) SD: 12.4	**0.004**
6 months	74.03 (34.2-100) SD: 14.1	75.72 (50-100) SD: 13.7	0.489
1 year	81.54 (47.5-100) SD: 14.6	81.94 (57.1-100) SD: 15.4	0.893
KSS Function			
Preoperative	60.33 (0-100) SD:16.3	55.10 (0-100) SD: 20.6	**0.042**
6 weeks	68.61 (0-100) SD: 18.1	60.41 (5-100) SD: 20.8	**0.004**
6 months	80.54 (20-100) SD: 16.3	73.21 (20-100) SD: 19.0	**0.012**
1 year	82.95 (10-100) SD: 16.7	74.64 (40-100) SD: 19.2	**0.017**
KSS Pain			
Preoperative	17.84 (0-50) SD: 12.3	17.84 (0-45) SD: 13.8	0.999
6 weeks	42.64 (10-50) SD: 11.7	41.94 (0-50) SD: 12.8	0.700
6 months	44.96 (10-50) SD: 9.8	47.44 (20-50) SD: 6.8	0.131
1 year	46.66 (10-50) SD: 7.7	44.29 (10-50) SD: 11.9	0.160
KSS pain motion			
Preoperative	49.06 (11-89) SD: 13.9	51.65 (16-92) SD: 19.3	0.250
6 weeks	83.84 (38-100) SD: 14.0	85.50 (45-100) SD: 14.8	0.457
6 months	88.35 (18-100) SD: 12.6	93.75 (50-100) SD: 9.6	**0.015**
1 year	90.90 (40-100) SD: 10.2	89.70 (50-100) SD: 14.9	0.593
KSS total composite			
Preoperative	109.03 (29-186) SD: 24.2	106.75 (28-179) SD: 33.5	0.560
6 weeks	152.59 (61-200) SD: 25.2	145.07 (83-200) SD: 26.7	0.063
6 months	168.91 (68-200) SD: 22.4	166.11 (102-200) SD: 22.4	0.489
1 year	173.61 (102-200) SD: 21.2	165.26 (106-200) SD: 28.7	0.068
FJS			
Preoperative	14.74 (0-100) SD: 15.9	11.91 (0-62.5) SD: 13.4	0.257
6 weeks	27.44 (0-97.9) SD: 22.6	29.42 (0-81.82) SD: 23.4	0.606
6 months	47.40 (0-100) SD: 27.8	51.33 (2.1-100) SD: 29.1	0.429
1 year	59.81 (0-100) SD: 28.5	72.36 (12.5-100) SD: 29.5	0.031*
Extension			
Preoperative	6.49 (-10-33) SD: 5.4	6.88 (0-25) SD: 6.9	0.650
6 weeks	4.03 (0-20) SD: 4.0	3.73 (0-20) SD: 4.2	0.630
6 months	1.69 (0-25) SD: 3.2	0.76 (0-15) SD: 2.6	0.090
1 year	0.85 (0-15) SD: 2.4	0.36 (0-5) SD: 1.3	0.285
Flexion			
Preoperative	117.81 (75-149) SD: 12.0	117.59 (75-145) SD: 12.7	0.906
6 weeks	117.67 (60-145) SD: 13.5	117.50 (90-143) SD: 11.8	0.934
6 months	127.51 (90-152) SD: 11.4	127.53 (105-143) SD: 8.2	0.994
1 year	130.71 (100-149) SD: 10.0	132.43 (117-145) SD: 7.2	0.382

FJS = Forgotten Joint Score, KOOS-JR = Knee Injury and Osteoarthritis Outcome Score, KSS = Knee Society Score, and SD = standard deviation.

Statistical significance was assessed using Student's *t*-test, with significant results shown in bold.

* An asterisk and bold outline denotes results that meet the threshold for clinical significance based on Minimal Clinically Important Difference standards.

**Fig. 3:**
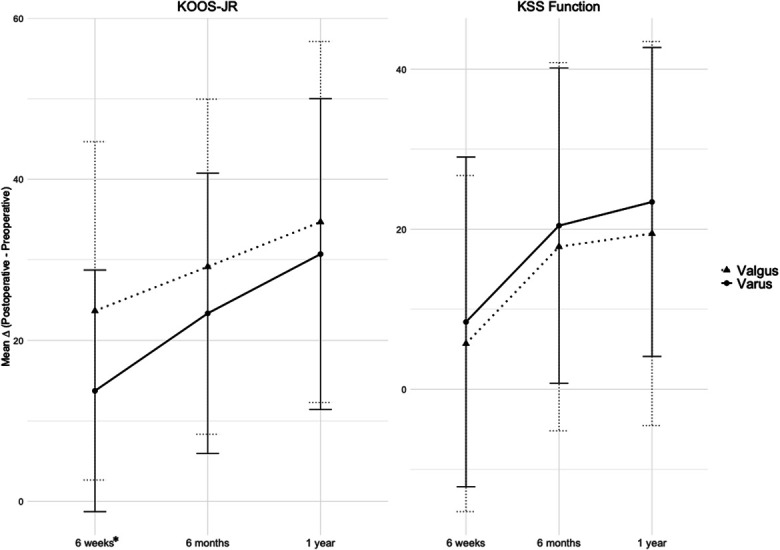
Mean improvement in KOOS-JR and KSS Function scores at 6 weeks, 6 months, and 1 year postoperative compared with preoperative baseline. Asterisks indicate statistically significant differences in the magnitude of improvement between varus and valgus groups at the corresponding time points. Error bars represent standard deviations. KOOS-JR = Knee Injury and Osteoarthritis Outcome Score, and KSS = Knee Society Score.

The improvement of KSS Function scores from baseline to each postoperative visit did not significantly differ between groups (p > 0.05). The valgus group demonstrated significantly greater improvement in KOOS-JR scores from preoperative to 6 weeks postoperatively (p ≤ 0.0001). At 6 months and 1 year postoperatively, the improvement was similar between groups (p = 0.06 and 0.32, respectively) (Fig. 3).

At latest follow-up, 1 patient in the varus group underwent revision for aseptic loosening, resulting in a survivorship of 99.7% (301/302). One additional patient in the varus group required manipulation under anesthesia (MUA) but did not require revision. No patients in the valgus group required revision or MUA.

## Discussion

This study supports the hypothesis that patients with preoperative valgus knee alignment undergoing KA/MP-TKA achieve equivalent outcomes to patients with varus alignment. Although the varus group demonstrated a higher KSS Function score postoperatively, the preoperative KSS Function score was also higher. The mean improvement from baseline to 1-year postoperatively was similar between groups (22.62 vs. 19.54, respectively), suggesting comparable functional gains.

The valgus group demonstrated greater improvement from baseline in KOOS-JR scores at 6 weeks postoperatively and better mean KSS Pain-Motion scores at 6 months. Unexpectedly, the valgus group demonstrated a better FJS at 1 year postoperatively than the varus group (72 vs. 60). This difference was statistically significant and clinically meaningful based on the Minimal Clinically Important Difference (MCID) threshold^31-33^. Valgus patients may experience greater preoperative impairment, as evidenced by lower preoperative KSS Function and KOOS-JR scores, and may be more appreciative of postoperative improvements, positively affecting their FJS scores. There were no other clinically important differences between the groups using established MCID thresholds^34-36^. One study has compared valgus and nonvalgus knees using KA-TKA with MP implants, reporting statistically better PROMs for nonvalgus knees, but the differences lacked clinical significance, and the angular thresholds defining the groups were quite different^5^.

In this study, the valgus group achieved a mean 1-year postoperative flexion (132°) up to 21° greater than literature values^37-40^. This may reflect the use of KA and/or MP implants. Unlike MA, which forces rectangular flexion gap symmetry, KA preserves physiologically asymmetric flexion gaps^41,42^. A retrospective analysis found that unrestricted KA-TKA frequently results in asymmetric flexion gaps without compromising functional outcomes^41^. A meta-analysis by Gao et al. found that KA results in better flexion than MA 6 to 24 months post-TKA^43^. The 1-year flexion of the valgus group in this study (132°) surpasses the postoperative weighted average flexion of MA studies (119°) reported in the meta-analysis by 13°—exceeding the Minimal Detectable Change threshold of 9.6°^43-45^. MP implants may also contribute to the flexion reported in the present study and have been associated with enhanced ROM^46-49^.

Concerns remain that KA may lead to residual postoperative valgus deformity, producing a less desirable cosmetic appearance and compromising longevity due to malalignment^3,9^. However, our valgus patients had an average postoperative HKA angle of 183.0°, dispelling the misconception that KA in valgus knees results in apparent postoperative deformities. In addition, a very limited number of subjects crossed over from 1 preoperative alignment group to the other group postoperatively.

Limitations of this study include the small valgus sample size—an issue shared by all comparisons of varus to valgus—however, our cohort reflects the expected 10% prevalence of valgus alignment, suggesting larger samples would likely yield similar results^1,2,5,50^. In addition, this is a single-surgeon series, which may introduce bias in patient recruitment, and the average preoperative ROM of 117° may be higher than typically encountered elsewhere. Furthermore, 1-year follow-up is insufficient to comment on longevity, although survivorship was high at latest follow-up, with one aseptic loosening in the varus group and none in the valgus group. We acknowledge that future studies should enroll more participants and follow them for 5 to 10 years to assess the longevity of KA and MP implants in valgus knees.

Another limitation involves varied definitions of valgus alignment across studies. Consistent with other literature and typical US patients, we defined valgus as HKA ≥183° and varus as HKA ≤177°^51,52^. We did not subcategorize patients because the cohort did not have individuals with severe alignment variations (>20°) or incompetent medial collateral ligaments, limiting the applicability of our analysis to more complex cases requiring higher constraint implants^2^. Thus, our findings may not extend to tertiary referral centers treating more severe deformities.

Finally, this study evaluates a combination of KA and MP implants, which may be unfamiliar to many surgeons and not reflective of standard practice^53^. Most reports document MA with non-MP implants, often involving variable surgical steps that are difficult to standardize^20^. Similar outcomes may be achievable with other implant designs, but the literature remains limited.

Still, the best outcomes for valgus knees are typically no better than outcomes for varus knees when MA is used^54^. By contrast, this study indicates that, by changing the alignment strategy and implant selection, clinical outcomes in valgus knees can be as good as or better than those in varus knees. Notably, we found a clinically superior 1-year FJS for the valgus group. These results address a gap in the literature by demonstrating the potential of this alternative treatment option.

## Conclusion

Patients with preoperative valgus knee alignment who undergo KA-TKA with true “ball-in-socket” MP implants achieve postoperative outcomes that are clinically equivalent or superior to those of patients with varus alignment. In this study, the valgus group demonstrated better outcome scores and flexion than those reported in the literature. These results support KA-TKA using MP implants as an appropriate surgical approach for preoperative valgus alignment, with added benefits of reduced invasiveness and complexity. However, the widespread adoption of this approach should await long-term data to verify that there is no increased rate of aseptic loosening.
